# Efficacy and Effectiveness of Exercise on Tender Points in Adults with Fibromyalgia: A Meta-Analysis of Randomized Controlled Trials

**DOI:** 10.1155/2011/125485

**Published:** 2011-10-09

**Authors:** George A. Kelley, Kristi S. Kelley, Dina L. Jones

**Affiliations:** ^1^Meta-Analytic Research Group, Robert C. Byrd Health Sciences Center, Department of Community Medicine, School of Medicine, West Virginia University, P.O. Box 9190, Morgantown, WV 26506-9190, USA; ^2^Department of Orthopaedics and Division of Physical Therapy, West Virginia University, Morgantown, WV 26506-9196, USA

## Abstract

Fibromyalgia is a major public health problem affecting an estimated 200 to 400 million people worldwide. The purpose of this study was to use the meta-analytic approach to determine the efficacy and effectiveness of randomized controlled exercise intervention trials (aerobic, strength training, or both) on tender points (TPs) in adults with fibromyalgia. Using random effects models and 95% confidence intervals (CI), a statistically significant reduction in TPs was observed based on per-protocol analyses (8 studies representing 322 participants) but not intention-to-treat analyses (5 studies representing 338 participants) (per-protocol, *g*, −0.68, 95% CI, −1.16, −0.20; intention-to-treat, *g*, −0.24, 95% CI, −0.62, 0.15). Changes were equivalent to relative reductions of 10.9% and 6.9%, respectively, for per-protocol and intention-to-treat analyses. It was concluded that exercise is efficacious for reducing TPs in women with FM. However, a need exists for additional well-designed and reported studies on this topic.

## 1. Introduction

 Fibromyalgia (FM), a chronic disorder characterized by widespread musculoskeletal pain, fatigue, and tenderness in localized areas, is a syndrome of unknown etiology. An estimated 200 to 400 million adults worldwide have FM with prevalence rates higher among women than men [1]. In addition to the personal consequences, substantial healthcare costs are accrued. For example, between 2002 and 2005, annual healthcare costs in the US were three times higher in people with FM versus those without FM [2]. 

 Management of FM includes both pharmacologic and nonpharmacologic approaches [3]. One nonpharmacologic approach is exercise, a low-cost intervention that is available to the vast majority of people with FM. A previous meta-analysis that included six studies published up to July of 2005, and in which one of the outcomes for widespread pain and tenderness was tender-points (TP) assessment, concluded that moderate-intensity aerobic exercise training probably leads to little or no difference in TP scores while strength training may result in large reductions in TP scores [4]. However, since that time, additional studies leading to conflicting results on this topic have been published and/or located [5–8]. In addition, while the authors preferentially analyzed intention-to-treat results, they also mixed these analyses with per-protocol results if data for the former were not available. This may be problematic, since each approach attempts to answer a different research question [9]. Furthermore, meta-analyses need to be updated on a regular basis [10]. Thus, the purpose of this study was to use the meta-analytic approach to determine the effects of exercise (aerobic, strength training, or both) on chronic widespread pain and tenderness using TP scores in adults with FM.

## 2. Methods

### 2.1. Data Sources

Studies for the current meta-analysis were retrieved from a large in-house exercise and rheumatic disease database that included 1024 citations after removing duplicates. This database was developed by searching six electronic sources (PubMed, EmBase, Cochrane Central Register of Controlled Clinical Trials, CINAHL, SPORTDiscus, and Dissertation Abstracts Online), cross-referencing from retrieved studies, including review articles, and expert review (Dr. Miriam Nelson, Tufts University, personal communication, June 13, 2008). All computer searches were conducted by the second author with the assistance of the first author. From the 1024 citations in the database, a search for studies dealing with the effects of exercise on widespread pain and tenderness, as defined by TP scores, in participants with FM was conducted using the single keyword “fibromyalgia” while searching across all indexed fields within the database. Detailed queries for original searches of each database are available upon request from the corresponding author.

### 2.2. Study Selection

The inclusion criteria for this study were (1) randomized controlled trials with the unit of assignment at the participant level, (2) an exercise intervention group (aerobic, strength training, or both), (3) exercise interventions ≥4 weeks in duration, (4) a comparative control group (nonintervention, usual care, and attention control), (5) adults ages 18 years and older with FM as defined by the American College of Rheumatology [11], (6) published and unpublished studies (master's theses and dissertations), (7) studies published in any language between January 1, 1980 and January 1, 2008, and (8) data available for TP scores [4]. All methods of assessing TP were included because they have been shown to correlate well with each other [12]. In addition, while provisional criteria from the American College of Rheumatology recently recommended that TP assessment be replaced with a combination of a wide pain index (WPI) and severity scale of symptoms (SS) score in the diagnosis of FM [13], past studies have relied primarily on TP versus WPI and SS outcomes. Furthermore, these new criteria are provisional in nature and have been criticized for replacing TP assessment in the diagnosis of FM [14].

### 2.3. Data Abstraction

 Codebooks for the abstraction of data were developed and included the following major categories: (1) study characteristics, (2) participant characteristics, (3) exercise intervention characteristics, (4) TP assessment characteristics, and (5) TP outcomes. All studies were coded by the first two authors, independent of each other. The authors then reviewed every item for correctness. Disagreements were resolved by consensus. Using Cohen's kappa statistic [15], the overall agreement rate prior to correcting discrepant items was 0.93, considered to be “almost perfect” [16].

### 2.4. Risk of Bias Assessment

The risk of bias assessment tool recently recommended by the Cochrane Collaboration was used to assess bias across six domains: (1) sequence generation, (2) allocation concealment, (3) blinding to group assignment, (4) incomplete outcome data, (5) selective outcome reporting, and (6) other potential bias [17]. Each domain was classified as having either a high, low, or unclear risk of bias [17]. The decision rule for blinding was that participants, research personnel, and outcome assessors were blinded to the primary outcome of interest, that is, changes in TP. Blinding of all groups was considered important given the subjective nature of TP measures. For other potential sources of bias, we included baseline differences in TP between the exercise and control groups as well as whether all subjects were reported as not participating in a regular exercise program, as defined by the authors, prior to taking part in the study. All assessments were conducted by the first two authors, independent of each other. Both authors then met and reviewed every item for agreement. Disagreements were resolved by consensus. Using Cohen's kappa statistic [15], the overall interrater agreement prior to correcting discrepant items was 0.62, considered to be “substantial” [16].

### 2.5. Statistical Analysis 

#### 2.5.1. Calculation of Study-Level Effect-Size Estimates for TP

The primary outcome of interest was changes in TP. Given the different metrics and reporting methods used, a standardized effect size (*g*) was calculated for all TP outcomes from each study [18]. This was accomplished by subtracting the difference in change scores between the exercise and control groups and then dividing by the pooled standard deviation of the change scores [18]. All *gs *were adjusted for small-sample bias [18]. To maintain independence, a pooled *g* was calculated for all studies that included multiple exercise groups and/or multiple TP measures, for example, tender point count and myalgic score, while keeping per-protocol and intention-to-treat results independent of each other. A negative *g* was indicative of improvement in TP.

#### 2.5.2. Pooled Estimates for TP

Random effects models that incorporate heterogeneity into the analysis were used to pool TP outcomes (*g*) from each study and were reported according to whether the data were analyzed using a per-protocol or intention-to-treat approach. In terms of magnitude, values for *g* of 0.20, 0.50, and 0.80 have been suggested to represent small, medium, and large effect sizes [19]. For *g*, two-tailed 95% confidence intervals (CI) that did not include zero (0) were considered to be statistically significant. In order to determine treatment effects on the whole distribution, that is, how treatment effects from new individual trials would be distributed about the mean, recently developed 95% prediction intervals (PI) for meta-analysis were calculated for effect size changes in TP [20, 21]. 

 Heterogeneity of TP outcomes between studies was examined using the *Q* statistic and a commonly used alpha value for statistical significance of 0.10 [18]. Consistency of between-study findings for TP outcomes were analyzed using *I* ^2^ [22]. Generally, *I* ^2^ values of 25%, 50%, and 75% may be considered to represent small, medium, and large amounts of inconsistency [22]. Small-study effects were examined using the regression approach of Egger et al. [23]. Small-study effects may be due to publication bias, selective reporting of outcomes [24, 25], true heterogeneity [23, 26], artifacts [27], or chance [28]. Two-tailed 95% confidence intervals that did not include zero (0) were considered to be suggestive of small-study effects. To examine the influence of each study on the pooled results, each study was deleted from the model once and the pooled analyses conducted with that one study deleted from the model. Cumulative meta-analysis, ranked by year, was also performed for the purpose of examining changes in TP over time [29].

#### 2.5.3. Moderator Analysis

Mixed effects models were used to examine potential between-group differences (*Q*_*b*_) when partitioned according to type of training (aerobic, strength training, or both), whether subjects were reported as sedentary prior to the intervention (yes versus unclear) and type of analysis (per-protocol versus intention-to-treat). An alpha level of ≤0.05 was considered to be indicative of a statistically significant between-group (*Q*_*b*_) difference. Because of the small number of studies included, as well as missing data for different variables from different studies, we did not attempt to conduct any additional moderator analyses.

#### 2.5.4. Regression Analyses

Simple, mixed effects meta-regression (method of moments approach) was used to determine the relationship between changes in TP and age in years, symptom years, and weeks of exercise training according to per-protocol and intention-to-treat analyses. Ninety-five percent CIs that did not include zero (0) were considered statistically significant. Because of the small number of studies included, as well as missing data for different variables from different studies, we did not attempt to conduct any additional meta-regression analyses.

#### 2.5.5. Other Analyses

Differences in baseline characteristics (age, symptoms, and diagnosis) were analyzed using the original metric (years). These were calculated by subtracting the baseline score in the exercise group from the baseline score in the control group. Variances were calculated from the pooled standard deviations of baseline scores in the exercise and control groups. In addition, differences in dropout rates between exercise and control groups were calculated using the risk difference (RD). Random-effects models were used to pool results for all analyses, while heterogeneity and inconsistency were examined using *Q* and *I* ^2^, respectively. 

#### 2.5.6. Data Reporting and Software

Data are reported as mean with 95% CI, 95% PI, mean ± standard deviation ($\overset{̅}{X}$ ± SD) and median (Mdn) with interquartile range (IQR). All data were analyzed using the metan routine [30] in Stata (version 11.0) [31], Comprehensive Meta-Analysis (version 2.2) [32], and PASW (version 18.0) [33]. 

## 3. Results

### 3.1. Study Characteristics

**Figure 1:**
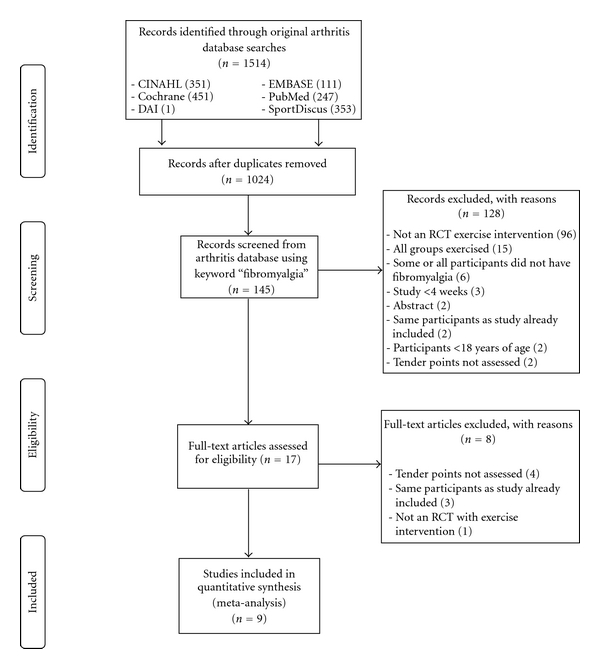
Flow diagram for the selection of studies. Stepwise procedures used for the selection of tender point studies. Note that RCT means randomized controlled trial.

Of the 1,024 studies screened, nine representing 19 groups (10 exercise and 9 control) and 362 participants (200 exercise and 162 control) met the criteria for inclusion [5–8, 34–38]. The number of exercise groups exceeded the number of studies, because one study included more than one exercise group [37]. A flow diagram that describes the search process is shown in Figure 1, while a general description of the characteristics of each included study is shown in Table 1. Two studies from a previous systematic review [4] were excluded because they did not meet all inclusion criteria. All of the studies were published in English-language journals between 1996 and 2007 [5–8, 34–38]. Three studies were conducted in Canada [34, 36, 37], two each in Finland [8, 35], and Spain [5, 7], and one each in Norway [38] and the United States [6]. Five studies reported using both per-protocol and intention-to-treat in the analysis of their data [6, 34, 36–38] but per-protocol data were not available for one of them [6]. Another three studies were limited to the per-protocol approach [5, 7, 35], while one reported no dropouts during the study [8]. All studies used a repeated measures design [5–8, 34–38]. With the exception of one study [8], all reported receiving funding for conducting their project [5–7, 34–38]. Only two studies included males [34, 38]. 

**Figure 2:**
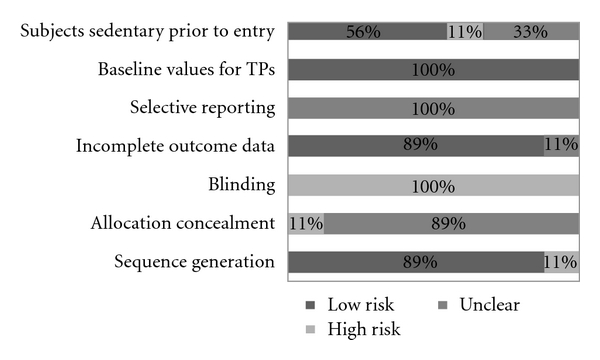
Risk of bias assessment. Percentage of studies classified as having a low, high, or unclear risk of bias for the seven listed categories. Note that TP means tender points.

 Results for risk of bias are shown in Figure 2. Across all studies, the risk of bias for blinding was high, while the risk for baseline differences in TP was low. For all studies, it was unclear whether there was bias for selective reporting of findings. With the exception of one study, the risk of bias for sequence generation and incomplete outcome data was considered low, while the risk of bias for allocation concealment was high. For one-third of the studies, the risk of bias for participants not being sedentary prior to study entry was unclear. 

### 3.2. Participant Characteristics

 A general description of the participants for each group from each study is provided in columns two through five of Table 1. The number of participants in the 10 exercise groups ranged from 8 to 30 ($\overset{̅}{X}\pm \text{SD}$ = 20 ± 8; Mdn = 18, IQR = 17), while the number of participants in the 9 control groups ranged from 10 to 31 ($\overset{̅}{X}\pm \text{SD}$ = 18 ± 7; Mdn = 17, IQR = 10). The majority of participants (98%) were female. The percentage of dropouts ranged from 0% to 46.7% in the exercise groups ($\overset{̅}{X}\pm \text{SD}$ = 22.2%  ± 18.9%; Mdn = 19%, IQR = 35%) and 0% to 63% in the control groups ($\overset{̅}{X}\pm \text{SD}$ = 13.6%  ± 21.4%; Mdn = 0%, IQR = 22%). A trend for a greater percentage of dropouts in the exercise versus control groups was found (RD = 7.9%, 95% CI, −0.001, 0.16, *Q* = 16.7, *p* = 0.05, *I* ^2^ = 46.2%, 95% CI, 0%, 74.2%). The mean between-group ages of participants, in years, ranged from 39 to 60 in the exercise groups and 37 to 59 in the controls. For those groups in which data were available, the mean between-group number of years reported for FM symptoms ranged from 7.8 to 24.0 in the exercise groups and from 7.0 to 19.0 in the controls, while the mean between-group number of years reported for the diagnosis of FM ranged from 2.8 to 7.6 in the exercise groups and from 3.6 to 7.6 in the controls. No statistically significant differences were found between the exercise and control groups in relation to baseline age, symptoms, or diagnosis of FM (Table 2). 

 Five exercise and six control groups reportedly took some type of prescribed drug for FM before and during the intervention period. These included muscle relaxants, antidepressants, anxiolytics, analgesics, nonsteroidal anti-inflammatory drugs, hypnotics, anticonvulsants, and sleep medications. For cigarette smoking, three groups (two exercise and one control) reported including some participants who smoked. Inadequate data were available for alcohol intake and diet. 

 Six exercise and five control groups were reported as being sedentary prior to taking part in the study. For menopausal status, eight of the ten exercise groups and seven of the nine control groups included both pre- and postmenopausal women, while one exercise and one control group included pre- and postmenopausal women only. In relation to overweight and obesity, 12 groups (six exercise and six control) reported that one or more participants were overweight or obese.

### 3.3. Exercise Intervention Characteristics

 A description of the exercise interventions from each study are shown in column 6 of Table 1. Five groups participated in aerobic exercise, three in strength training, and two in both. For the eight groups that reported data, six reported that the exercise sessions were supervised, while two reported that they were unsupervised. Length of training ranged from 12 to 23 weeks ($\overset{̅}{X}\pm \text{SD}$ = 16 ± 4; Mdn = 16, IQR = 9), while mean between-group frequency ranged from two to seven sessions per week ($\overset{̅}{X}\pm \text{SD}$ = 3 ± 1; Mdn = 3, IQR = 1). For the four intervention groups in which adequate data were available, mean between-group compliance, defined as the percentage of exercise sessions attended, ranged from 67% to 97% ($\overset{̅}{X}\pm \text{SD}$ = 83.2 ± 14.6; Mdn = 85, IQR = 27). For those groups that participated in aerobic exercise, the mean between-group duration of exercise in the four groups for which adequate data were provided ranged from 12 to 25 minutes per session ($\overset{̅}{X}\pm \text{SD}$ = 19 ± 5; Mdn = 20, IQR = 9). Within-group intensity of aerobic training ranged from 50% to 80% of maximum heart rate for the six groups in which data were available, while the duration of training ranged from five to 40 minutes per session. Five of the seven groups that participated in aerobic exercise included exercise in the water, either alone or in conjunction with land-based exercise. For the five groups that included strength training, the within-group range was one to four sets (three groups reporting), one to 20 repetitions (five groups reporting), six to 11 exercises (four groups reporting), and 40% to 80% of 1-repetition maximum (three groups reporting).

### 3.4. TP Assessment Characteristics

A description of the methods used for TP assessment is shown in the last column of Table 1. Two [6, 37] of the nine [5–8, 34–38] studies included multiple measures for TP assessment, while three reported using a dolorimeter [7, 37, 38]. Two studies included a myalgic score [7, 37], and four reported that the outcome assessor was blinded to group assignment [6, 34, 36, 37]. 

### 3.5. TP Outcomes 

#### 3.5.1. Per-Protocol Analysis

**Figure 3:**
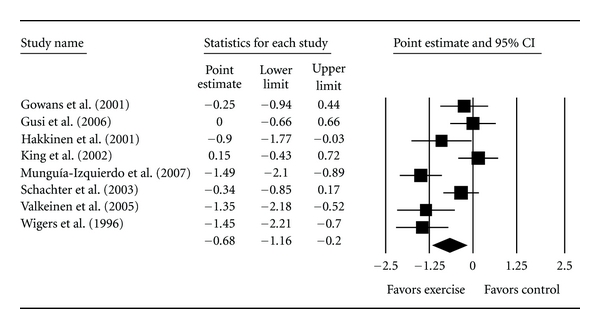
Forest plot for changes in tender points according to per-protocol analysis. The black squares represent the standardized mean difference (Hedge's *g*), while the left and right extremes of the squares represent the corresponding 95% confidence intervals. The middle of the black diamond represents the overall standardized mean difference (Hedge's *g*), while the left and right extremes of the diamond represent the corresponding 95% confidence intervals.

Eight studies representing 322 participants (186 exercise and 146 control) were included in the final assessment of TP using the per-protocol approach [5, 7, 8, 34–38]. Overall, a statistically significant reduction in TP was found (Table 3 and Figure 3). This was equivalent to a relative reduction of 10.9%. A statistically significant amount of heterogeneity was observed as well as a large amount of inconsistency. The 95% PI for a new trial was from −2.25 to 0.89. No statistically significant small-study effects were observed (−5.1, 95% CI, −15.1, 4.9). With each study deleted from the model once, results remained statistically significant across all deletions. Cumulative meta-analysis, ranked by year, showed that TP results have remained statistically significant since 2005. 

 When a moderator analysis was conducted according to type of training, statistically significant reductions in TP were limited to strength training with no within-group heterogeneity (Table 3). Reductions in TP congruent with strength training were equivalent to a relative reduction of 12.2%. However, no statistically significant between-group differences were observed for the three types of training (*Q*_*b*_ = 2.6, *p* = 0.28). For those studies in which participants were reported as sedentary prior to enrollment, a statistically significant reduction of approximately 8.2% was found. However, statistically significant heterogeneity was observed as well as a large amount of inconsistency. No statistically significant between-group differences were found (*Q*_*b*_ = 0.004, *p* = 0.95). Meta-regression resulted in no statistically significant association between age (−0.02, 95% CI, −0.11, 0.05), symptom years (0.01, 95% CI, −0.09, 0.12), and length of training (−0.03, 95% CI, −0.16, 0.10).

#### 3.5.2. Intention-to-Treat Analysis

**Figure 4:**
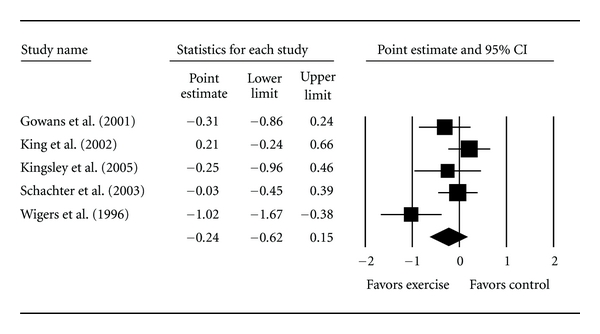
Forest plot for changes in tender points according to intention-to-treat analysis. The black squares represent the standardized mean difference (Hedge's *g*), while the left and right extremes of the squares represent the corresponding 95% confidence intervals. The middle of the black diamond represents the overall standardized mean difference (Hedge's *g*), while the left and right extremes of the diamond represent the corresponding 95% confidence intervals.

Five studies representing 338 participants (211 exercise, 127 control) were included in pre- and post assessment of TP using the intention-to-treat approach [6, 34, 36–38]. Overall, no statistically significant reduction in TP was found (Table 3 and Figure 4). This was equivalent to a relative reduction of 6.9%. A statistically significant amount of heterogeneity was observed as well as a moderate amount of inconsistency. When compared to per-protocol results no statistically significant between-group differences were observed (*Q*_*b*_ = 2.0, *p* = 0.16). The 95% PI for a new trial was −1.48 to 1.0. No statistically significant small-study effects were observed (−5.2, 95% CI, −14.4, 4.0). With each study deleted from the model once, results remained nonsignificant across all deletions. Cumulative meta-analysis, ranked by year, showed that results have been nonsignificant since 2001. As can be seen in Table 3, no statistically significant within or between-group differences were found when intention-to-treat results were partitioned according to type of training (*Q*_*b*_ = 0.0, *p* = 0.99) or whether participants were sedentary prior to enrollment (*Q*_*b*_ = 0.22, *p* = 0.64). Meta-regression resulted in no statistically significant association between age (0.06, 95% CI, −0.25, 0.39), symptom years (−0.39, 95% CI, −0.96, 0.17), or length of training (−0.01, 95% CI, −0.12, 0.09).

## 4. Discussion

 The purpose of the current meta-analysis was to examine the effects of exercise on TP in adults with FM. The overall per-protocol results, significant since 2005 as well as with each study deleted from the model once, suggest that exercise is efficacious for improving TP in selected women with FM. In other words, potentially important benefits can be derived for those who comply with the exercise intervention [39]. However, it is not known whether the observed relative reduction of 11% is practically important. Consequently, other nonpharmacologic and/or pharmacologic interventions may be necessary [40]. Regardless, exercise should almost always be recommended because of the numerous other benefits that can be derived from such [41], including those specific to adults with FM [42]. Along those lines, it would appear prudent to recommend that women with FM adhere to the general exercise guidelines recommended by others [43–45].

 While a significant within-group reduction in TP for strength training was observed, there were no between-group differences when compared to aerobic exercise or combined aerobic and strength training. In addition, results for strength training were based on only two outcomes. Furthermore, given that studies are not randomly assigned to predictors, moderator and meta-regression analyses are considered to be observational in nature [46]. Consequently, such analyses do not support causal inferences [46]. Finally, moderator and meta-regression analyses in aggregate data meta-analysis tend to be underpowered as well as being subjected to potential confounding and ecological bias [47]. Clearly, the validity of the current per-protocol findings needs to be tested in large, well-designed randomized controlled trials. 

 The lack of statistically significant findings for intention-to-treat analyses suggest that overall, exercise is not effective for reducing TP scores in women with FM. In other words, when the results of participants who drop out of an exercise intervention are pooled with those who do not drop out, the overall positive effect of exercise across all participants is no longer significant [39]. This has important implications when recommending various interventions such as exercise in the prevention and treatment of disease. However, the lack of statistically significant findings based on intention-to-treat analysis may have been the result of a lack of statistical power for the current meta-analysis. For example, post hoc power analysis using previously developed methods for meta-analysis [48] resulted in a power of 0.44 for intention-to-treat findings. 

 Future randomized controlled exercise intervention studies in adults with FM could improve on the reporting of several variables. Based on our risk of bias assessment, future studies should make sure to report the protocol number for their study so that one can determine whether selective outcome reporting occurred. In addition, information on whether the participants were sedentary prior to taking part in the study is important since the effects of exercise may not be fully realized if the participants had been exercising prior to enrollment. Given the subjective nature of TP assessment, information on blinding of the participant, outcome assessor, and other relevant personnel is also needed. However, the risk of bias for blinding may always be high given the difficulty in blinding participants to the exercise intervention. Future studies need to also do a better job in providing complete information on the number of years since the diagnosis of FM, diet intake, including alcohol, as well as any therapies, pharmacologic or nonpharmacologic, in which the participants are engaged. Finally, complete information on the exercise interventions should be reported. For aerobic exercise, this includes the length, frequency, intensity and duration of exercise as well as the training modality, compliance to the exercise protocol, equipment used, if any, and setting in which exercise took place. For strength training, this includes the length, frequency, intensity and duration of exercise, number of sets, repetitions and exercises, rest period between exercises, equipment used, if any, as well as compliance to the exercise protocol and setting in which exercise took place. 

 Several suggestions regarding future studies appear appropriate. For example, while the prevalence of FM is greater in women than in men [49], it is recommended that future studies include more men in randomized controlled exercise intervention studies. This is especially true given that 98% of the participants included in the current meta-analysis were women. Given the current emphasis on dose response [50], future randomized controlled trials should also include different exercise training regimens in order to determine the optimal exercise program or programs for adults with FM.

 The major strength of the current meta-analysis is the reporting of separate results according to per-protocol and intention-to-treat analyses, thus allowing one to determine whether the treatment works (per-protocol analysis) as well as whether it works in the real world (intention-to-treat analysis) [39]. While the results of the current meta-analysis provide important, updated information in relation to the efficacy and effectiveness of exercise on TP scores in participants with FM, these findings need to be viewed with respect to the following potential limitations beyond those previously mentioned. First, a moderate-to-large amount of heterogeneity and inconsistency was observed for our TP results. Given these findings and despite the fact that a random-effects model that incorporates heterogeneity into the analysis was used, the generalization of such results may not be appropriate [17]. However, the use of such statistics to determine true heterogeneity and inconsistency is rather arbitrary in nature, and thus, should be viewed with caution [51]. A second potential limitation is the fact that the prediction intervals for estimating the expected results of a new trial included zero for TP outcomes. However, these values should not be confused with confidence intervals since prediction intervals are based on a random mean effect while confidence intervals are not [20, 21]. A third potential limitation is the large number of statistical tests that were conducted. As a result, some of the significant findings could have been nothing more than chance findings. However, adjustments for multiple tests were not made because of the more severe problems associated with such [52, 53]. 

## 5. Conclusions

 The results of the current meta-analysis suggest that exercise is efficacious for reducing TP scores in selected women with FM. These findings are important, because they provide support for the use of exercise for decreasing widespread pain and tenderness in women who exercise on a regular basis. However, a need exists for additional well-designed and reported studies on this topic, especially those that examine the effectiveness (intention-to-treat approach) of exercise on TP scores in men and women with FM.

## Figures and Tables

**Table 1:** General characteristics of included studies.

Reference	*N*	Age (Years)	Gender (F/M)	FM symptoms (Years)	Exercise intervention	Tender point assessment
Gowans et al. [34]	Ex: 27 Con: 23	Ex: 44.6 ± 8.7 Con: 49.8 ± 7.3	F (88%)/M F (87%)/M	Ex: 9.6 ± 8.6 Con: 8.4 ± 7.6	23 weeks supervised, facility-based aerobic exercise, 3x/wk, 20 min/day, 60%–75% MHR; compliance, 67%.	Sum of up to 18 TP; assessor blinded to group assignment

Gusi et al. [5]	Ex: 17 Con: 17	Ex: 51 ± 10 Con: 51 ± 9	F F	Ex: 24 ± 9 Con: 19 ± 8	12 weeks supervised pool-based exercise, 3x/wk; aerobic (20 min/day, 65%–75% MHR, strengthening (20 min/day, 4 sets, 10 reps); compliance >94%	Sum of up to 18 TP

Hakkinen et al. [35]	Ex: 11 Con: 10	Ex: 39 ± 6 Con: 37 ± 5	F F	Ex: 12 ± 4 Con: 12 ± 10	17 weeks supervised strength training, 6–8 ex, 2x/wk, 5–20 reps, 40%–80% 1RM	Sum of up to 18 TP

King et al. [36]	Ex: 46 Con: 39	Ex: 45.2 ± 9.4 Con: 47.3 ± 7.3	F F	Ex: 7.8 ± 6.1 Con: 9.6 ± 7.9	12 weeks supervised, facility-based aerobic ex, 3x/wk, 10–40 min/day, 75% MHR	Sum of up to 18 TP; both assessors blinded to group assignment

Kingsley et al. [6]	Ex: 15 Con: 14	Ex: 45 ± 9 Con: 47 ± 4	F F	Ex: 9 ± 10 Con: 7 ± 5	12 weeks strength training, 11 ex, 2x/wk, 1 set, 8–12 reps, 40%–80% 1RM	Total number of TP and total myalgic score; assessor blinded to group assignment

Munguía-Izquierdo and Legaz-Arrese [7]	Ex: 29 Con: 24	Ex: 50 ± 7 Con: 46 ± 8	F F	Ex: 14 ± 10 Con: 14 ± 9	16 weeks supervised, facility-based ex, 3x/wk; strengthening (1–3 sets, 8–15 reps, 8–10 ex); aerobic (20–30 min, 50%–80% MHR); compliance ≥75%	Sum of up to 18 TP

Schachter et al. [37]	Ex (sb): 56 Ex: (lb): 51 Con: 36	Ex (sb): 41.9 ± 8.6 Ex: (lb): 41.3 ± 8.7 Con: 42.5 ± 6.7	F F F	Ex (sb): 8.6 ± 6.0 Ex (lb): 8.8 ± 6.2 Con: 8.8 ± 5.0	16 weeks home-based, low-impact aerobic ex; short bout, 2x/day, 3x/wk, 5–15 min/session, 40%–75% HRR; long bout, 1x/day, 3x/wk, 10–30 min/session, 40%–75% HRR	Mean number of TP and mean myalgic score using a dolorimeter; assessor blinded to group assignment

Valkeinen et al. [8]	Ex: 13 Con: 13	Ex: 60 ± 2 Con: 59 ± 4	F F	NR	21 weeks supervised strength training, 6–7 ex, 2x/wk, 5–20 reps, 40%–80% 1RM, 97% compliance	Sum of up to 18 TP

Wigers et al. [38]	Ex: 20 Con: 20	Ex: 43 ± 9 Con: 46 ± 9	F (90%)/M F (95%)/M	Ex: 9.0 ± 5 Con: 11 ± 9	14 weeks supervised aerobic exercise, 3x/wk, 18–20 min/day, 60%–70% MHR	Mean number of TP using dolorimetry

Notes: Description of groups and subjects from each study limited to those that met the inclusion criteria; *N*, Initial number of subjects as reported by authors; age reported as mean ($\overset{̅}{X}$) ± standard deviation (SD); F, females; M, males; Ex, Exercise; Con, Control; FM, fibromyalgia; MHR, maximum heart rate; 1RM, one-repetition maximum; HRR, heart rate reserve; lb, long bout; sb, short bout; min, minutes; wk, week; reps, repetitions; TP, tender points; NR, not reported.

**Table 2:** Baseline characteristics of exercise and control groups.

	Exercise			Control			Difference	Heterogeneity	Inconsistency
Variable	*N*	$\overset{̅}{X}\pm \text{SD}$	Mdn (IQR)	*N*	$\overset{̅}{X}\pm \text{SD}$	Mdn (IQR)	$\overset{̅}{X}$ (95% CI)	*Q* (*p*)	*I* ^2^ (95% CI)
Age (years)	10	46.1 ± 6.1	45 (9)	9	47.3 ± 6.0	47 (6)	−0.5 (−2.1, 1.0)	13.5 (0.14)	33.1% (0, 68.1)
FM Symptoms (years)	8	11.4 ± 5.1	9 (4)	8	11.2 ± 3.8	10 (5)	0.04 (−1.2, 1.2)	5.1 (0.65)	0% (0, 56.0%)
FM Diagnosis (years)	4	4.2 ± 2.3	3 (4)	3	5.1 ± 2.2	4 (4)	−0.5 (−1.3, 0.3)	1.4 (0.71)	0% (0%, 72.0%)

Notes: FM, fibromyalgia syndrome; *N*, number of groups reporting data; $\overset{̅}{X}\pm \text{SD}$, mean ± standard deviation; Mdn (IQR), median and interquartile range; $\overset{̅}{X}$ (95% CI), mean and 95% confidence intervals; *Q* (*p*), heterogeneity statistic and alpha value; *I* ^2^ (95% CI), percent inconsistency and 95% confidence interval.

**Table 3:** TP results.

Variable	Studies (#)	*g* (95% CI)	*Q* (*p*)	*I* ^2^ (95% CI)
PP Analysis				
Overall	8	−0.68 (−1.16, −0.20)*	28.6 (<0.0001)**	75.5% (50.9%, 87.8%)
Type of training				
Aerobic	4	−0.44 (−1.04, 0.16)	11.2 (0.01)**	73.2% (24.7%, 50.5%)
Strength	2	−1.13 (−1.73, −0.53)*	0.5 (0.47)	0%
Both	2	−0.75 (−2.22, 0.71)	10.8 (0.001)**	90.7% (66.7%, 97.4%)
Sedentary prior to enrollment				
Yes	5	−0.67 (−1.23, −0.08)*	16.6 (0.002)**	76.0% (41.2%, 90.2%)
Unclear	3	−0.71 (−1.73, 0.31)	11.8 (0.003)**	83.1% (48.6%, 94.4%)

ITT Analysis				
Overall	4	−0.24 (−0.62, 0.15)	10.1 (0.04)**	60.6% (0%, 85.2%)
Type of training				
Aerobic	4	−0.24 (−0.71, 0.22)	10.1 (0.02)**	70.3% (37.4%, 93.7%)
Strength	1	0.25 (−0.96, 0.46)	—	—
Both	—	—	—	—
Sedentary prior to enrollment				
Yes	2	−0.14 (−0.47, 0.20)	0.62 (0.43)	0%
Unclear	3	−0.33 (−1.1, 0.42)	9.4 (0.009)**	78.8% (32.2%, 93.4%)

Notes: PP, per-protocol; ITT, intention-to-treat; *g* (95% CI), Hedge's *g* and 95% confidence intervals; *Q* (*p*), heterogeneity statistic and alpha value; *I* ^2^ (95% CI), percent inconsistency and 95% confidence interval; —, insufficient data to calculate; *statistically significant within-group difference because 95% confidence intervals do not include 0; **statistically significant heterogeneity (*p* ≤ 0.10).
